# Early high-resolution immune profiles are associated with survival, relapse and graft-versus-host-disease after allogeneic hematopoietic cell transplantation

**DOI:** 10.1038/s41409-026-02901-5

**Published:** 2026-05-30

**Authors:** Patrick Terrence Brooks, Lia Minculescu, Hans Jakob Hartling, Rebecca Svanberg Teglgaard, Jose Antonio Salado-Jimena, Lone Smidstrup Friis, Brian Kornblit, Ida Schjødt, Søren Lykke Petersen, Niels Smedegaard Andersen, Jens Lundgren, Susanne Dam Nielsen, Lars Klingen Gjærde, Hanne Vibeke Marquart, Henrik Sengeløv, Sisse Rye Ostrowski

**Affiliations:** 1 Department of Clinical Immunology, Copenhagen University Hospital—Rigshospitalet, Copenhagen, Denmark; 2 Department of Hematology, Copenhagen University Hospital—Rigshospitalet, Copenhagen, Denmark; 3 Department of Infectious Diseases, Copenhagen University Hospital—Rigshospitalet, Copenhagen, Denmark; 4 Department of Clinical Medicine, Faculty of Health and Medical Sciences, University of Copenhagen, Copenhagen, Denmark

**Keywords:** Translational research, Bone marrow transplantation

## Abstract

Allogeneic hematopoietic cell transplantation (allo-HCT) is a well-established treatment in several hematological malignancies, where post-transplant complications are largely immune-driven. Post-transplantation (post-HCT) immune status assessment is essential for clinical monitoring of patients. In this study, immune cell composition and function was evaluated in whole blood from 77 adult allo-HCT recipients taken day +28 post-transplantation. High-resolution immunophenotyping assessed innate and adaptive immune compartments, including activation and immune checkpoint markers, while cytokine release after stimulation with T cell or innate stimuli examined immune function. Improved survival was associated with increased immune cell counts of both innate and adaptive immune compartments, as well as elevated cytokine-release responses. Elevated myeloid and innate lymphoid subsets and innately stimulated cytokine release were also associated with lower relapse incidence. Increased CD4 T cell PD-1 expression and HLA-DR on CD8 T cells was associated with poorer overall survival. These findings emphasize the importance of early innate immune reconstitution, indicating that integrating functional and phenotypic profiling could reveal new information regarding cell subsets of interest for allo-HCT patient monitoring, as well as potential targets for immunomodulation or cell therapies.

## Introduction

Allogeneic hematopoietic cell transplantation (allo-HCT) is a key treatment for hematological and immune-driven diseases [1, 2]. After transplantation, donor-derived hematopoietic cells replace recipient production of bone marrow function, while immune cells stemming from the graft exert an active role in eradicating residual leukemic cells through graft-versus-leukemia (GvL) effects in malignant conditions [3–5]. While allo-HCT clinical outcomes have improved significantly, patient mortality and clinical complications, i.e., relapse, and acute graft-versus-host-disease (aGVHD) remain major concerns [6–8]. Many post-transplantation complications are immune-driven [9, 10]. Therefore, immune-based assessment methods, i.e., flow cytometric immune phenotyping, hematological cell counts, and donor/recipient chimerism analyses may support clinical decision-making in allo-HCT [11, 12].

Identification of early prognostic biomarkers in allo-HCT remains under active investigation [13–15]. While high-resolution immunophenotyping has been used to investigate associations with post-transplantation (post-HCT) clinical outcomes [16, 17], multimodal immune profiling, including functional stimulated cytokine release combined with immunophenotyping or transcriptional profiling, has also been used to examine differences between patient subgroups and associations with clinical outcomes such as infectious complications [18–21]. However, the complex relationship between post-HCT immune cell composition and function warrants further investigation to substantiate the potential clinical value of functional and cellular immune profiles as early allo-HCT biomarkers of clinical outcomes including overall survival, relapse, and aGVHD.

In this study, high-resolution immunophenotyping investigating composition and activation status of major immune cell compartments was combined with functional assessment using whole blood stimulated cytokine release, as well as post-HCT T cell chimerism. These immune profile markers, measured at day +28 post-transplantation, were examined for associations with clinical outcomes of allo-HCT.

## Methods

### Study design and clinical outcomes

**Fig. 1:**
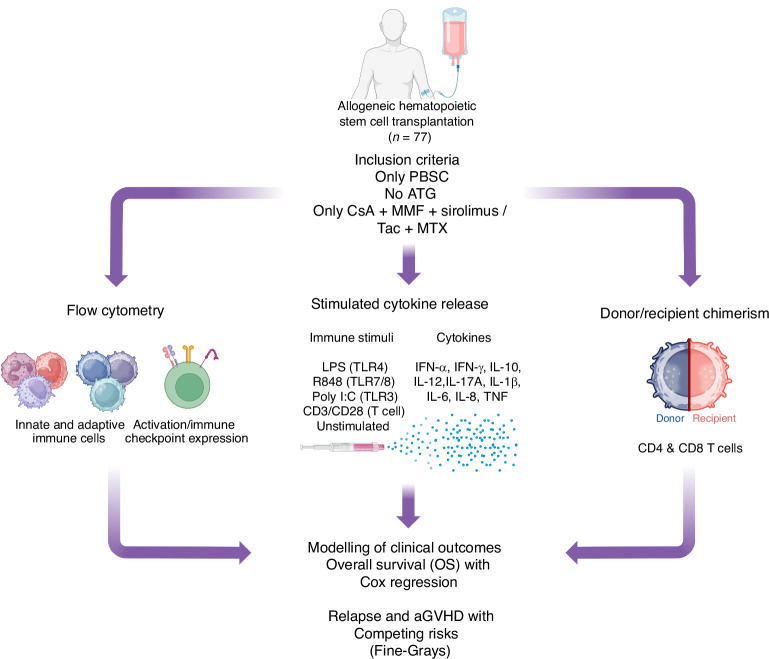
Study setup. aGVHD acute graft-versus-host disease, ATG anti-thymocyte globulin, CD cluster of differentiation, CD3/CD28 T-cell activation via CD3 and CD28, IFN interferon, IL interleukin, LPS lipopolysaccharide, OS overall survival, PBSC peripheral blood stem cells, Poly I:C polyinosinic:polycytidylic acid, R848 resiquimod, TLR toll-like receptor, TLR3 toll-like receptor 3, TLR4 toll-like receptor 4, TLR7/8 toll-like receptors 7 and 8, TNF tumor necrosis factor. (Created in BioRender. Ostrowski, S. (2026) https://BioRender.com/ol6dl7a).

We previously reported on the current cohort of 77 adult patients who received allo-HCT for a hematological malignancy. Patient inclusion was from March 2021 to July 2023 [21]. Immunoprofiling of fresh whole blood samples obtained at day +28 post-HCT combined (1) a standardized, customized, and clinically implemented flow cytometry panel, specially developed for immune cell profiling of immune dysfunction/-dysregulation in lymphoid and myeloid compartments, (2) a standardized functional assay, quantifying cytokine release in response to four immune-stimuli, and an unstimulated control, and (3) day +28 CD4 and CD8 T cell chimerism assessment (Fig. 1). Incidence of relapse, aGVHD (grade II-IV) and overall survival were included as clinical endpoints. The study was approved by the Data Protection Agency (RH-2015-04, I-Suite nr: 03605) and Danish Ethical Committee (H-17024315) and conducted in accordance with the Declaration of Helsinki. All patients gave written and oral informed consent before inclusion in the study.

### Patient characteristics

Patient characteristics are summarized in Table 1. Median age was 63 years (interquartile range [IQR], 55–68 years) and 61% were male. The most common allo-HCT indications were myelodysplastic syndrome (52%) and acute myeloid leukemia (31%). Thirty-four patients received myeloablative (MA) conditioning with fludarabine and treosulfan (FluTreo), and 43 patients received non-myeloablative (NMA, FluTBI-2-3Gy). Peripheral blood stem cell (PBSC) apheresis was the only graft-source. At time-of-transplantation, 22 AML patients were in 1st complete remission (CR), one in 2nd CR, while another had primary induction failure. Of two acute lymphoblastic leukemia patients, one was in 1st CR, and one had unknown status. Among seven lymphoma patients, five were in CR and two had missing data. Donor median age was 26 years (IQR: 22–34 years), and 36% were female with 10 female-to-male (FTM) transplantations. Most donors were 10/10 human leukocyte antigen (HLA) matched unrelated donors (MUD) (81%). Conditioning-related GVHD-prophylactics: NMA—cyclosporine A (CsA) + mycophenolate mofetil (MMF) + sirolimus; MA—tacrolimus (Tac) + methotrexate (MTX). Conditioning regimens dosages found in Supplementary Materials.

**Table 1:** Patient characteristics.

	All, *n* = 77		MA, *n* = 34		NMA, *n* = 43	
Age, years median (IQR)	63	(55–68)	58	(52–64)	65	(61–70)
Sex, n (%)						
Female	30	(39.0)	15	(44)	15	(35)
Male	47	(61.0)	19	(56)	28	(65)
Disease, n (%)						
Myelodysplastic syndrome	40	(52)	23	(68)	17	(40)
Acute myeloid leukemia	24	(31)	9	(27)	15	(35)
Lymphoma	9	(12)	0	(0.0)	9	(21)
Acute lymphoblastic leukemia	2	(2.6)	1	(2.9)	1	(2.3)
Other leukemia	2	(2.6)	1	(2.9)	1	(2.3)
Conditioning, n (%)						
Myeloablative	34	(44)	34	(100)	0	(0.0)
Non-myeloablative	43	(55)	0	(0.0)	43	(100)
Conditioning regimen, n (%)						
FluTreo	34	(44)	34	(100)	0	(0.0)
FluTBI-2/3 Gy	43	(56)	0	(0.0)	43	(100)
Karnofsky Performance Status, n (%)						
<80	9	(12)	5	(15)	4	(9.3)
90	41	(53)	16	(47)	25	(58)
100	24	(31)	13	(38)	11	(26)
Unknown	3	(3.9)	0	(0.0)	3	(7.0)
Donor age, years median (IQR)	26	(22–34)	27	(22–36)	26	(22–34)
Donor sex, female, n (%)	28	(36)	15	(44)	13	(30)
Female donor/male recipient (FTM), n (%)	10	(13)	5	(15)	5	(12)
Recipient/donor relation, n (%)						
Unrelated	69	(90)	27	(79)	42	(98)
Sibling	8	(10)	7	(21)	1	(2.3)
Recipient/donor HLA match grade, n (%)						
MUD	62	(81)	26	(77)	36	(84)
MMUD	8	(10)	1	(2.9)	7	(16)
MSD	7	(9.1)	7	(21)	0	(0.0)
Graft-versus-host-disease prophylaxis, n (%)						
CsA + MMF + sirolimus	43	(56)	0	(0.0)	43	(100)
Tac + MTX	34	(44)	34	(100)	0	(0.0)

*ALL* acute lymphoblastic leukemia, *AML* acute myeloid leukemia, *CsA* cyclosporine A, *FluTBI-2/3* *Gy* fludarabine plus total body irradiation 2–3 Gy, *FluTreo* fludarabine plus treosulfan, *HLA* human leukocyte antigen, *IQR* interquartile range, *MA* myeloablative, *MMF* mycophenolate mofetil, *MMUD* mismatched unrelated donor, *MSD* matched sibling donor, *MTX* methotrexate, *MUD* matched unrelated donor, *MDS* myelodysplastic syndrome, *NMA* non-myeloablative, *Tac* tacrolimus.

### High-resolution immunophenotyping

High-resolution immunophenotyping was performed on EDTA whole blood within 24 hours of sampling using previously described, customized, antibody panels (DuraClone, Beckman Coulter, USA), comprising a 10-color flow cytometric immune profiling tool. The gating strategies for the panels, that have previously been reported in detail [22], were designed to assess innate and adaptive immune compartments including activation and immune checkpoint expression. Expression levels of activation and immune checkpoint markers were reported as median-fluorescence intensity (MFI). Antibody panels included a known number of added acquisition beads allowing absolute counts of immune subsets. Immune phenotype definitions and markers shown in Supplementary information file 2 tables SX1.

### Assessment of stimulated cytokine release

Heparinized whole blood was incubated for 22 h in commercially available immune stimulation TruCulture tubes (Rules-Based Medicine, USA). Four immune-stimuli (CD3/CD28 T cell-stimulation; lipopolysaccharide (LPS), toll-like-receptor-4 (TLR4)-stimulation; resiquimod (R848) TLR7/8-stimulation, polyinosinic-polycytidylic acid (poly I:C), and TLR3-stimulation) and an unstimulated tube were applied. After incubation, TruCulture supernatants were removed and stored. Cytokine concentration analysis of IFN-α, IFN-γ, IL-10, IL-12(IL-12p40), IL-17A, IL-1β, IL-6, IL-8, and TNF used Luminex technology (Luminex, USA) as previously described [22]. Studies of innate immune cells, such as phagocytes, demonstrate a pronounced post-stimulation imbalance between IL-12 isoforms, with elevated IL-12p40 and low IL-12p70 production [23], supporting the selection of IL-12p40. As IL-12p40 is a shared subunit between IL-12/IL-23, its measurement may also reflect IL-23-related responses.

### Post-HCT CD4/CD8 chimerism

Donor/recipient-chimerism data on CD4 and/or CD8 T cells analyzed within ±5 days of day +28 sampling, was collected from electronic health records at the Department of Clinical Immunology where available. Chimerism analysis applied short tandem repeats used until March 2022; hereafter, analyses used next-generation-sequencing as previously described [24].

### Statistical analyses

Cox proportional hazard regression models assessed associations between overall survival and log-transformed immune cell counts, stimulated cytokine release, and activation/immune checkpoint marker expression. MFI was not log-transformed. Overall survival results were reported as hazard ratios (HR). Multivariate models were adjusted for conditioning regimen, age, and donor-recipient match-grade. Univariate Cox regression of patient characteristics reported in Supplementary information file 2 tables SX2. Cox model p-values were adjusted for multiple comparisons using the Benjamini-Hochberg method [25] (adjusted significance-threshold: <0.05). For relapse and aGVHD, associations were estimated using Fine-Grays competing risk models. Non-relapse mortality was the competing risk for relapse, while non-relapse mortality and relapse were competing risks for aGVHD. Four patients experienced aGVHD before sampling and were excluded from aGVHD-analyses. Statistical analyses were done with R version 4.3.2.

## Results

### Patient clinical outcomes

During the follow-up period (median: 774 days; IQR: 593–1028; range: 434–1216 days), 14 patients experienced relapse. Grade II-IV aGVHD was diagnosed in 13 patients, where 4 patients had developed aGVHD before day 28 post-HCT. For 19 patients who died during follow-up, 11 deaths were attributed to relapse.

### Associations of immune cell profiles and CD4/CD8 chimerism with overall survival

**Fig. 2:**
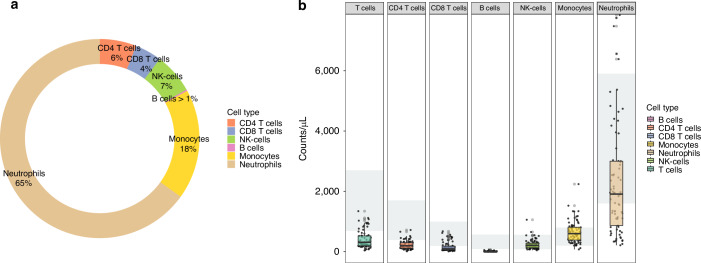
Distribution plot of major immune cell lineages. ** a** Donut plot of immune cell  distributions and **b** boxplots of immune cell counts at day +28 after allogeneic hematopoietic cell transplantation. Gray rectangles represent normal reference ranges (ranges shown in Supplementary information file 2 Table SX3).

High-resolution immune cell profiling by flow cytometry at day +28 post-HCT assessed major immune cell subsets including immune checkpoint and activation marker expression. By day +28, innate immune cell counts appeared normalized: neutrophils were at the lower reference limit, monocytes were normal or slightly elevated, and NK cells were within normal range (Fig. 2). In contrast, within the adaptive compartment, T cells, including both CD4/CD8 subsets, and especially B cells, remained markedly reduced. Post-HCT cell counts shown in Supplementary information file 2 Table S1.

**Fig. 3:**
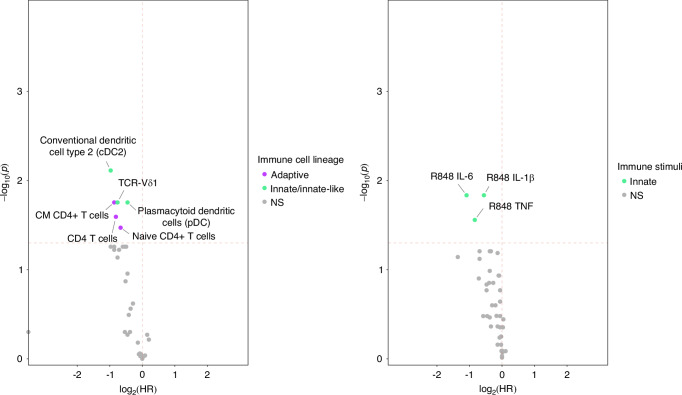
Volcano plots of hazard ratios (HR) from Cox regression models for overall survival (*N* = 77). Left pane: Immune cell subset counts. Right pane: stimulated cytokine release. The x-axis shows the log2 value of the hazard ratio (log2HR), while the y-axis shows the negative log10 of the *p* values (-log10(*p* value)) for each cox regression model. Only variables with a statistically significant result after BH p-value adjustment are labeled. Horizontal, red-dotted lines denote FDR (BH) adjusted alpha-value threshold = 0.05. CM central memory, EM effector memory, HR hazard ratio, cDC2  type 2 conventional dendritic cells, NK cell natural killer cell, NS not significant, TCRαβ alpha-beta T cell receptor.

Multiple post-HCT immune subsets were positively associated with improved overall survival. Among adaptive immune cells, higher counts of total CD4 T cells (HR 0.57, 95% CI 0.40–0.83; BH-adjusted *p* = 0.025), naive CD4 T cells (HR 0.63, 95% CI 0.46–0.87; BH *p* = 0.034), and central memory (T_CM_) CD4 T cells (HR 0.55, 95% CI 0.38–0.80; BH-adjusted *p* = 0.018) were associated with lower mortality. Similarly, TCR-Vδ1 γδ T cells (HR 0.59, 95% CI 0.43–0.83; BH-adjusted *p* = 0.018) also demonstrated a protective association. Amongst innate subsets, higher counts of type 2 conventional dendritic cells (cDC2; HR 0.51, 95% CI 0.35–0.73; BH-adjusted *p* = 0.008) and plasmacytoid dendritic cells (pDC; HR 0.73, 95% CI 0.60–0.88; BH-adjusted *p* = 0.018) were associated with improved survival (Fig. 3, Supplementary information file 2, Table S2). HRs ranged from 0.51 to 0.73, indicating a protective effect of higher cell counts. Other adaptive and innate/innate-like cell types trended in univariate analyses (FDR-adjusted) but lacked significance in multivariate models. In conditioning-based survival analyses, significant associations persisted for cDC2s (HR 0.50, 95% CI 0.34–0.73; BH-adjusted *p* = 0.01) and pDCs (HR 0.65, 95% CI 0.51–0.83; BH-adjusted *p* = 0.01) in NMA-conditioned patients while higher counts of αβ T cells, including CD4/CD8 subsets, and γδ T cells in these patients, showed trends toward protective effects.

Assessing post-HCT immune activation marker expression revealed that elevated levels of immune checkpoint marker PD-1 on CD4 T cells (HR 2.72, 95% CI 1.41, 5.24; BH-adjusted *p* = 0.045) and activation marker HLA-DR on CD8 T cells (HR 1.22, 95% CI 1.09, 1.35; BH-adjusted *p* = 0.01) were associated with increased mortality (Supplementary information file 2 tables S3). CD4 and/or CD8 T cell donor/recipient chimerism was available for 65 patients. No associations between chimerism and overall survival was observed for CD4 (HR 1.00; 95%CI 0.97, 1.03; *p* = 0.86) or CD8 (HR 0.99; 95%CI 0.96, 1.03; *p* = 0.71) lineages, nor did conditioning-based sub analyses find associations (data not shown).

### Associations between stimulated cytokine release profiles and overall survival

For all patients, higher post-HCT R848-stimulated IL-1β, IL-6 and TNFα were the only cytokines associated with improved overall survival after FDR-adjustment (Fig. 3, Supplementary information file 2 tables S4). For NMA-conditioned patients, higher CD3/CD28-stimulated IFNγ, IL-17A, IL-1β, IL-8, and TNFα, R848-stimulated IFNα, IFNγ, IL-1β, IL-6, and TNFα, and LPS-stimulated IL-8, were associated with improved survival outcomes. No associations were found for MA-conditioning subgroup analyses, nor for Poly I:C stimulated release or unstimulated cytokine concentrations. Post-HCT stimulated cytokine release data shown in Supplementary Fig. S01.

### Associations between immunophenotypes and overall survival using Kaplan-Meier analysis

**Fig. 4:**
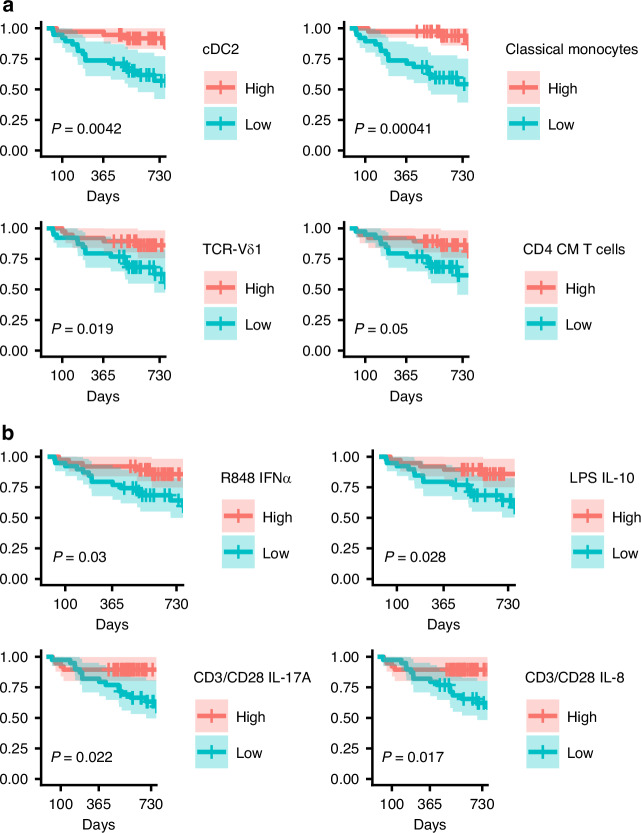
Kaplan-Meier plots of overall survival after allo-HCT according to median-split immune cell subsets and stimulated cytokine release. **a** Type 2 conventional dendritic cells (cDC2s), classical monocytes, TCR-Vδ1 γδ-T cells, and central memory CD4 T cells. **b** R848-stimulated IFNα, LPS-stimulated IL-10, and CD3/CD28-stimulated IL-17A and IL-8. Red lines and overlay denote high-level groups including 95% CI, while blue denotes low-level groups.

Kaplan-Meier curves of groups stratified by median-split immune cell counts (Fig. 4a) showed that higher cDC2, classical monocyte, TCRγδ-Vδ1 T cell, and CD4 T_CM_ counts were associated with improved overall survival. Early survival curve separation was notable for cDC2s and monocytes. Higher levels of distinct cytokines after LPS-, R848-, and CD3/CD28-stimulation were likewise associated with improved overall survival (Fig. 4b).

### Associations between immune profiles and relapse incidence

Higher incidence of relapse was observed in patients with lower counts of total monocytes (*p* = 0.005), cDC2 (*p* = 0.02), and monocytic myeloid-derived suppressor cells (mMDSC, *p* = 0.02)(Supplementary information file 2 tables S5). Additionally, increased expression of the activation markers HLA-DR (*p* = 0.02) and CD49d (*p* = 0.02) on classical monocytes was associated with increased incidence of relapse (Supplementary information file 2 tables S6). Lower cytokine release levels of CD3/CD28-stimulated IL-17A (*p* = 0.04) represented the only functional marker associated with higher relapse incidence (Supplementary information file 2 tables S7). However, in sub analyses of NMA-conditioned patients, lower levels of R848-stimulated IFNγ (*p* = 0.03) and IL-12 (*p* = 0.02), and Poly I:C-stimulated IL-12 (*p* = 0.02), were additionally found associated with higher incidence of relapse. CD4 and CD8 T cell chimerism showed no association with relapse incidence.

### Associations between immune profiles and aGVHD incidence

Higher aGVHD incidence was only observed to be associated with increased CD35 expression on non-classical monocytes (*p* = 0.01). No associations between immune cell counts, stimulated cytokine release, chimerism, and aGVHD were observed (Supplementary information file 2 tables S8–S10).

## Discussion

High-resolution immune profiling early after allo-HCT revealed that higher counts of innate and adaptive immune cell subsets, and improved immune function represented by higher levels of stimulated cytokine release, were positively associated with improved overall survival. Additionally, higher counts of specific myeloid and innate lymphoid subsets, as well as increased levels of innately stimulated cytokine release, were associated with a reduced incidence of relapse. The findings could indicate that in absence of a functional post-HCT adaptive T cell compartment capable of orchestrating essential immune functions such as GvL responses, innate immune cells of both myeloid and lymphoid lineages may assume a significant role in controlling the underlying disease in the early post-transplant period. The observed importance of innate cell counts, and functional innate-driven responses could, apart from influences from alloreactive, inflammatory and infectious processes, reflect trained innate immune responses previously noted in autoimmune disease, solid-organ transplantation, and allo-HCT [26, 27].

Reduced immune function at day 28 post-HCT could reflect immune dysfunction associated with increased post-HCT mortality. The concurrent positive associations between innate stimulation via TLRs, innate cell counts, and overall survival indicates the importance of functioning dendritic, innate-like lymphoid cells i.e. γδ T cells, and monocytic populations early after allo-HCT. We previously found associations between post-HCT monocyte counts and several innate-stimulated cytokines after adjusting for conditioning regimen in the same cohort, where immune cell counts alone could not fully account for functional response variability, and MA and NMA patients displayed distinct immune cell/immune function correlation profiles between subgroups [21]. Furthermore, we previously observed significant positive correlations between monocyte counts and total, CD4, and CD8 T-cell counts in this cohort, implicating coordinated regulation of innate and adaptive immunity during early immune reconstitution, consistent with adult and pediatric allo-HCT studies showing improved adaptive recovery with early innate recovery [28, 29]. Consistent with these prior observations, the current associations with improved survival outcome, observed also for NMA-conditioned patients, further supports that functional immune profiling could provide clinically relevant insights into post-HCT immune states.

Consistent with our observations linking lower post-HCT TCR γδ1 T cell counts to mortality and relapse, this innate-like T cell population has previously been associated with improved overall survival and a lower risk of relapse in a separate allo-HCT cohort from the same patient center [17]. Innate-like T cells [30] include γδ T cells, mucosal-associated invariant T cells (MAIT), and natural killer T cells (NKT), the latter showing a univariate association with overall survival in our results. These cell types have been implicated in both antibacterial and anticancer defenses [31], and γδ T cells also display antitumor activity largely independent of tumor mutational burden and MHC class I presentation, making them promising candidates for allogeneic therapies [32]. Also, incubation of γδ T cells with TLR7-stimuli has been shown to enhance γδ T cell-mediated cancer cell cytotoxicity [33].

Interestingly, elevated CD4 T cell counts, including naïve and T_CM_ subtypes of CD4 cells in all patients, as well as increased CD3/CD28-stimulated IFNγ, IL-12, IL-17A, IL-8 and TNF in NMA-conditioned patients, was associated with improved overall survival. This also supports that combined timely T cell-compartment reconstitution and T cell functionality are essential factors in immune recovery. The associations between increased T_CM_ counts and an improved overall survival also aligns with memory T cells being essential in immune reconstitution, and, while participating in alloreactive processes such as GvL and aGVHD, they also play a joint protective role against infections in coordination with innate immune cells [34–36].

In line with the associations between lower monocyte counts and relapse, day +28 monocytes were previously linked with improved overall survival and reduced risk of relapse after allo-HCT [37, 38]. Similarly, monocyte counts measured later during the post-HCT period have been associated with improved survival [39–41]. The association between higher mMDSC counts and a lower risk of relapse suggests a role for mMDSCs in modulating disease recurrence. It was previously demonstrated that AML and ALL patients with higher mMDSC levels pre-transplant, but not post-transplant, had an increased risk of relapse [42]. Furthermore, mMDSC have been implicated as GvL effectors [43]. mMDSC may support post-HCT immune regulation through myeloid compensation for impaired regulatory T cell function and may also have potential as a cell-based therapy for aGVHD [44, 45].

The associations observed between OS and early cDC2 and pDC counts, as well as increased relapse with lower cDC2 counts, are in line with known effects of dendritic cell types on post-HCT clinical outcomes [46, 47]. Graft and pDC counts have been linked with increased relapse and mortality, possibly due to the tolerogenic effects of pDC on GvL responses. Dendritic cell involvement in antineoplastic responses have also been relatively well characterized where cDC2 are known to prime CD4 T cells against cancer cells [48].

Post-HCT CD4 and CD8 immune checkpoint marker expression was associated with overall survival, but not with relapse or aGVHD. Increased expression of PD-1 on CD4 cells and HLA-DR on CD8 cells was associated with higher mortality, suggesting T cell exhaustion as a therapeutic target for post-HCT immunomodulation. In support of this, higher numbers of exhausted T cells found in the bone marrow were previously associated with increased relapse [49]. Also, additional potential T-cell targets associated with relapse i.e. TIGIT and CD161 on both CD4 T cells and innate-like T cells are emerging [50, 51]. Associations between mortality and T cell inhibitory targets have also been observed in patients with both myeloid and lymphoid primary conditions who relapsed after allo-HCT [52, 53]. However, increased incidence of aGVHD after pre-transplantation anti-PD-1 treatment is a concern in lymphoma patients undergoing allo-HCT [52, 54, 55]. Increased expression of HLA-DR and CD49d on classical monocytes was associated with an increased risk of relapse, whereas an increased CD35 expression on non-classical monocytes was associated with increased risk of aGVHD, suggesting that post-HCT myeloid cell activation could be used as predictors of these clinical allo-HCT outcomes [56, 57].

This study would have benefited from additional sampling timepoints to better assess the dynamics of immune cell and functional immune reconstitution in relation to clinical outcomes of allo-HCT. The positive findings linking cellular and functional biomarkers with clinical outcomes should be interpreted cautiously in the context of the relatively small sample size. Such results may be vulnerable to overestimation of effect sizes i.e. the winner’s curse, and may not replicate in larger, independent cohorts [58]. In the current study, the relatively low number of cases of aGVHD and relapse limited the power of conditioning-related sub analyses.

Effects from infection-related complications i.e. CMV-reactivation or other infections requiring intervention, important factors for allo-HCT outcomes and immune reconstitution [59], were not included in this study. While a broad range of immune cells and cytokine/stimuli-combinations were assessed, additional cell types, stimuli, and markers, also including plasma-based biomarkers, could provide more comprehensive high-resolution immunoprofiling. Incorporating direct or indirect measurements of tissue-resident cell types implicated in pathogenic processes after allo-HCT, e.g. innate lymphoid cells (ILCs) [56] or T stem cell memory (T_SCM_) cells [57], could also provide valuable insights.

In conclusion, parallel high-resolution immunophenotyping and functional assessment provided valuable novel insights into the early immune state after allo-HCT and its associations with clinical outcomes. Our findings highlight a critical role for early immune reconstitution of key immune subsets as well as immune function, in particular within the innate myeloid and lymphoid immune compartments, that, if further linked to underlying immune states preceding clinical complications, could improve clinical decision-making or support new cell-based therapies.

## Supplementary information

Supplementary materials

Supplementary information file 2

## Data Availability

Due to Danish privacy regulations, clinical data used in this study are only available from the corresponding author upon reasonable request.
