# Supplementary material for: Early high-resolution immune profiles are associated with survival, relapse and graft-versus-host-disease after allogeneic hematopoietic cell transplantation
Source: Bone Marrow Transplant. 2026 May 30;61(8):1006–14. doi: 10.1038/s41409-026-02901-5 (PMC13437226; doi:10.1038/s41409-026-02901-5)
Supplement: Supplementary file 1 [file 41409_2026_2901_MOESM1_ESM.docx]

**Supplementary Materials**

Myeloablative conditioning regimens combined fludarabine (150 mg/m²) + treosulfan (42 g/m²) (FluTreo). FluTreo is considered a reduced toxicity conditioning regimen. The non-myeloablative conditioning regimens combined fludarabine (90 mg/m2) and 2–3 Gy TBI (FluTBI-2Gy), or fludarabine (90 mg/m2) and 3 Gy TBI (FluTBI-3 Gy).

**Supplementary Materials Figure S01**: Day 28 post-HCT stimulated cytokine release concentrations. Grey boxes represent normal ranges for 31 heathy controls.
